# Plant a seed series: the impact of an online outreach package on school pupils’ knowledge, skills, and attitudes to medicine

**DOI:** 10.1186/s12909-024-05300-w

**Published:** 2024-03-29

**Authors:** Nadin Hawwash, Enam Haque

**Affiliations:** 1 School of Medical Sciences, Faculty of Biology, Medicine and Health, University of Manchester, Manchester, UK; 2 Cancer Research UK Manchester Cancer Research Centre, Manchester, UK

**Keywords:** Medicine, Outreach, PAS, Widening participation, Widening access

## Abstract

**Background:**

Medicine is one of the most inaccessible professions in the United Kingdom (UK). The Plant a Seed (PAS) series was created to address this; it is an online pre-recorded three-part video series to “Inspire”, “Educate” and “Motivate” pupils from widening participation backgrounds on Medicine. We explored the impact of PAS on pupils’ knowledge, skills and attitude to Medicine.

**Methods:**

We conducted a national pretest-posttest study of Years 7–9 pupils in the UK. 503 schools were invited to PAS, following ethics approval. Consented pupils viewed all three episodes asynchronously and completed a pre-and post-series Likert scale confidence questionnaire, which evaluated their knowledge, skills and attitudes to a medical career. A Shapiro-Wilk test showed the lack of a normal distribution (*p* < 0.05); therefore, a Wilcoxon signed-rank test statistically compared pretest-posttest results of each pupil.

**Results:**

70 pupils in Years 7–9 from 2 schools participated in this study. PAS was shown to significantly increase pupils’ knowledge of the role and life of a doctor, medicine as a degree, admissions requirements, and careers in medicine (*p* < 0.05). There was a significant increase in pupils believing they could study medicine. The intervention did not significantly increase the desire for pupils to study medicine (*p* = 0.187).

**Conclusion:**

PAS significantly improved pupils’ knowledge, skills and confidence demonstrating the need and benefit to enrolment of the programme at scale. It did not significantly increase the number of pupils wishing to study medicine. Analysis at scale is required to evaluate the effectiveness of PAS as a key intervention to break down barriers to medicine.

## Background

Medicine is one of the most inaccessible professions to enter given the application demands, financial requirements and limited opportunities available to students from working-class backgrounds [1]. According to the 2016 Social Mobility in the UK Report, just 4% of doctors in the UK come from a working-class background [2]. The 2013 General Medical Council (GMC)’s National Training Survey found that only 25% of medical trainees were from state schools, only 8% had received free school meals and only 6% grew up in the most deprived areas of the UK [3]. It is evident there is a longstanding skew in medicine towards more socio-economically advantaged pupils. Although most medical schools in the UK seek triple A A-level results, factors primarily considered beyond this which may disadvantage students from working-class backgrounds include the requirement for high achieving scores in the University Clinical Aptitude Test (UCAT) or BioMedical Admissions Test (BMAT), evidence of volunteering over a specific period in a healthcare setting and evidence of undertaking extra-curricular activities [4, 5]. Pupils may not be aware of such requirements generally or in a timely manner to allow for preparation. Pupils from working-class backgrounds may not have support networks within or outside of their academic institution to raise awareness on application requirements or provide support, guidance and opportunities [6]. Additionally, such preparation or engagement may have associated costs that not all students can afford, for example, a 6-day Medical Summer school costing £3,249 or online admissions preparation packages costing £595 [7, 8].

Widening participation (WP) aims to address the underrepresentation in medicine. WP describes attempts to increase pupils entering higher education, particularly from under-represented groups such as (i) low-income backgrounds, (ii) pupils from schools with low performance compared to the national average, (iii) from areas with limited higher education participation and (iv) first-generation higher education students [9]. Attempts to diversify medicine included the establishment of the National Medical Schools WP Forum (National Forum) in 2015, an organisation of 24 UK medical schools. A key highlight of their work was developing UKWPMed, a collaborative initiative consisting of 6 medical schools providing WP students aspiring to study medicine with an automatic interview and contextual offer in any of the partner institutions [10]. The National Forum identified a challenge to delivering WP initiatives was the lack of sustainable funding, which was needed for reimbursing travel costs and food expenses for pupils to attend outreach activities [10]. It made sense, therefore, to develop an WP initiative delivered at a minimal cost.

In the UK national curriculum, Key Stage 3 is defined as Years 7–9 and occurs prior to GCSE exams sittings in Year 10 and 11 and A-level sittings are in Year 12 and 13 [11]. WP initiatives in medicine mainly focus on primary school pupils (Year 1–6) or pupils in Year 9 to Year 13 [12–14]; however, we hypothesised that Years 7–9 was a critical age for raising awareness of medicine as a career as it was the timepoint when pupils consider choosing their GCSE subjects. In the UK, medical schools determine admission to their programmes based on the subjects chosen and grades obtained by applicants. For instance, a pivotal decision to be made during Year 7–9 for medicine includes studying the three sciences Chemistry, Biology and Physics separately as opposed to the BTEC qualifications whereby the latter limits the number of Medical Schools the student may be eligible to apply for. On applying to medicine, evidencing transferrable skills such as communication, leadership, teamwork and problem solving is essential as these are skills required during in the medical programme and for a career as a doctor [15]. Communication skills such as empathy, gathering information and listening are crucial skills to practice and use as a doctor to create a positive doctor-patient relationship to aid the provision of the best patient care [16].

A study by Martin et al. (2018) demonstrated that Year 9 pupils had minimal awareness of medicine and the breadth of medical careers [14]. McHarg et al. (2007), conducted semi-structured interviews with 15 medical students who felt that school support and raising their aspirations helped pupils from WP backgrounds to be motivated to apply to medicine [17]. McHarg et al. (2007) also found early exposure to medicine to be an important aspect in WP allowing time for the idea to “flourish” [17]. Gore et al. (2018) highlighted the importance of creating sustained outreach in primary and secondary schools [18].

The Plant a Seed (PAS) series, a low-cost sustained secondary school outreach package for Year 7–9 pupils, was created to address these points and provide early insight into medical school and medical careers. PAS is an online pre-recorded three-part video series, with each video mapping to one of the themes, “Inspire”, “Educate” and “Motivate”. Pupils had opportunity to view the three sessions over the academic year and thus had the time to reflect after each video. The low cost associated with running this fully asynchronous programme at scale highlights its sustainability. The PAS series shares similarities with the Social Cognitive Theory established by Albert Bandura in the 1960s. Bandura’s Social Cognitive Theory highlights the influence of interactions between personal factors such as knowledge and attitudes, environmental factors such as access in community and behaviours such as skills and self-efficacy [19, 20]. Consequently, we hypothesise that having the opportunity to observe and learn from positive role models through the PAS series will impact on a pupil’s knowledge, attitude and career aspirations thereby enhancing their self-efficacy, inspire and motivate them to consider medicine as a viable career prospect [19]. This study aimed to assess the impact the PAS online outreach package had on the knowledge, skills and attitude to Medicine as a degree and career of pupils in Years 7–9 from WP backgrounds.

## Methods

### Participants

To be eligible for this study, pupils had to (i) be in Years 7–9 at a secondary school in the UK, (ii) from a secondary school in a WP-flagged location and (iii) have completed the whole PAS series.

### Recruitment

We performed a national pretest-posttest study of PAS. We recruited UK secondary school pupils in Years 7–9, by emailing 503 schools located in WP postcode areas and inviting them to take part in the study. This information was obtained using the Higher Education Access Tracker (HEAT) database [21]. Schools that expressed interest were provided with an in-depth letter explaining the study and the PAS series. Schools committed to participating were then sent a link to the PAS webpage which included pupil assent forms, participant information sheets and parent consent forms. Schools that posted back completed forms for each participating pupil were recruited to the study. Printed forms and pre-paid postage envelopes were provided on request. Eligible schools were invited between June 2022 and December 2022 to complete the PAS series in their own time from the date they enrol onto the series till April 2023. Almost a year was provided to complete the PAS series to ensure the PAS sessions were conducted at 3 points over the academic year at times most suitable for schools that would not impact on learning the school curriculum or preparing for exams. This study was conducted as a series to allow time for consolidation of the knowledge gained from each part in the series. Pupils that did not provide signed consent and assent forms could not partake in the PAS series. Teachers were advised to arrange for alternative education sessions during school hours for pupils that did not provide consent, to ensure their education was not impacted by not participating in the series.

### PAS series

Three PAS video sessions were available in the PAS series. Each PAS video session lasted 30 to 60 min and began with a clip of the day in the life of a doctor or surgeon. The “Inspire” recording began with the day in the life of a GP, followed by the history of heart surgery and the development of vaccines. These topics aimed to inspire pupils to consider medicine as a career. The “Educate” session began with the day in a life of a foundation year doctor followed by the teaching on the anatomy of the lungs, as well as the pathophysiology, diagnosis, and treatment of asthma. This was developed to provide insight into the learning undertaken by medical students. The “Motivate” video looked at a day in a life of a surgeon, followed by the journey of a medical student through higher education. This was to make pupils believe they could follow a similar journey. All the PAS content including videos and worksheets, questionnaires, consent and participant information forms were validated by the University of Manchester ethics committee and Access and Success Team to ensure easy language was used that was accessible to all pupils. Additionally, all videos contained subtitles to ensure accessibility to pupils that are deaf or hard of hearing.

### Study design

Teachers were invited to watch a briefing video before starting the series, which explained how to navigate the videos and the specific tasks for pupils to complete in the workbook. Teachers then asked pupils to complete the pre-test study paper questionnaire with the pupil’s name on it before they started watching the first video. Teachers were asked to securely store the paper questionnaires in a secure place, to maintain confidentiality. They then accessed the PAS videos through a secure website link, hosted by the University of Manchester. This was accessible to them throughout the study period. Teachers downloaded the worksheets for each session and then handed them to pupils to complete during each session. After viewing all three videos in the PAS series, pupils were handed back their pre-study questionnaire. Pupils were instructed to first input their pre-study paper questionnaire results onto the online version of the questionnaire and then complete the post-test study questionnaire. This enabled the anonymous collection and pairing of pre-and post-series questionnaire feedback from the same pupil. Pupils were made aware in the participant information form that questionnaires submitted would be anonymous and that paper questionnaires would be destroyed.

### Questionnaire

The PAS questionnaire consisted of 11 questions with a five-point Likert scale (“Strongly Agree” to “Strongly Disagree”) that measured pupil confidence in knowledge, skills and attitudes towards medicine as a degree and career (Table 1). There was a paired analysis of pre-and post-study results for each pupil. Pre- and post-series questionnaires were identical except for the additional inclusion of a comments section. Pupils could complete this section to provide written feedback on the question “If you feel more informed about a career and how to study medicine, what has changed and why?”.

**Table 1:** The plant a seed series pre- and post-series questionnaire

Knowledge*
I know what a doctor does
I know about the medicine degree
I know about life as a doctor
I know about the different careers in medicine
I know about what is needed to get into medical school
I know the subjects I need to take to do medicine
I know about access Manchester
Skills*
I feel I have the necessary study skills to apply to medicine
I feel I have the necessary communication skills to study medicine
Attitude*
I want to study medicine
I feel confident in applying to medical school

*All questions were rated on a 5-point Likert scale (strongly agree, agree, undecided, disagree and strongly disagree).

### Statistical analysis

A Shapiro-Wilk test was used to assess whether there was a normal distribution in the data, and therefore the type of statistical analysis to use. Given the lack of a normal distribution (*p* < 0.05), a Wilcoxon signed-rank test was used to statistically compared pre- and post-series questionnaire results for each pupil to identify any significant differences in knowledge, skills and attitudes on attending the series.

## Results

### Knowledge

**Fig. 1:**
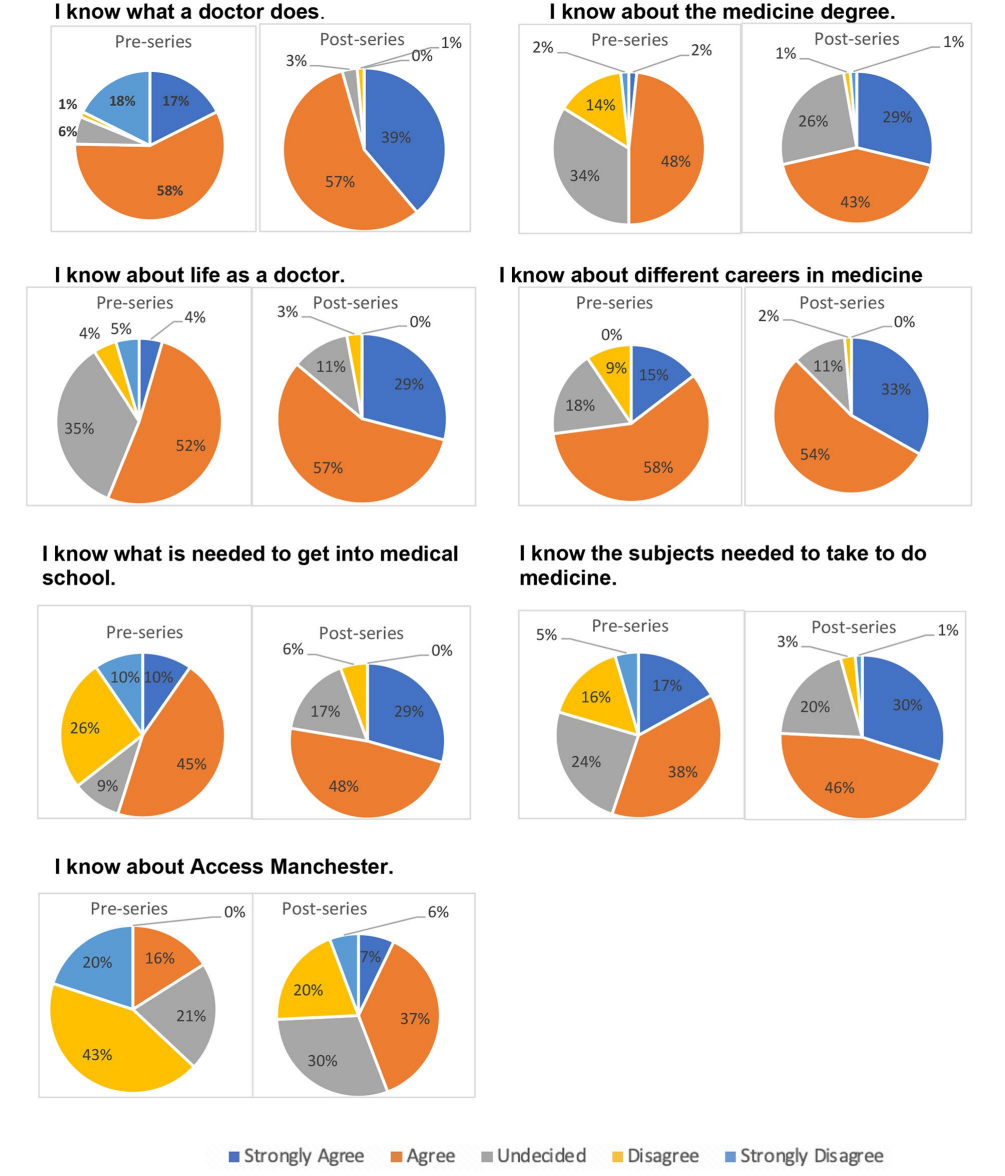
Pupil knowledge of medicine as a degree and career pre- and post-series

70 pupils in Years 7–9 from two participating schools located in Derbyshire were included in this study. On completion of the PAS series, there was a significant increase in pupils’ knowledge of what a doctor did (*p* = 0.002). The proportion of pupils that strongly agreed to the increase in their knowledge of what a doctor did rose from 17% before the session, to 39% after the session (Fig. 1). Pupils’ knowledge of medicine as a degree significantly increased (*p* < 0.0001). Overall, 2% of pupils strongly agreed that they know about the medicine degree pre-series which increased to 29% post-series (Fig. 1). Additionally, a significant increase was found in the knowledge of medicine undergraduate admissions requirements (*p* < 0.0001), as well as subject entry requirements (*p* < 0.0001). The PAS series also increased the knowledge in pupils’ understanding of life as a doctor (*p* < 0.0001). The proportion of pupils strongly agreeing to knowing about life as a doctor increased from 4% pre-series to 29% post-series. Furthermore, the proportion of pupils strongly agreeing with their awareness of the different careers in medicine more than doubled upon completion of the PAS series (Fig. 1).

### Skills

**Fig. 2:**
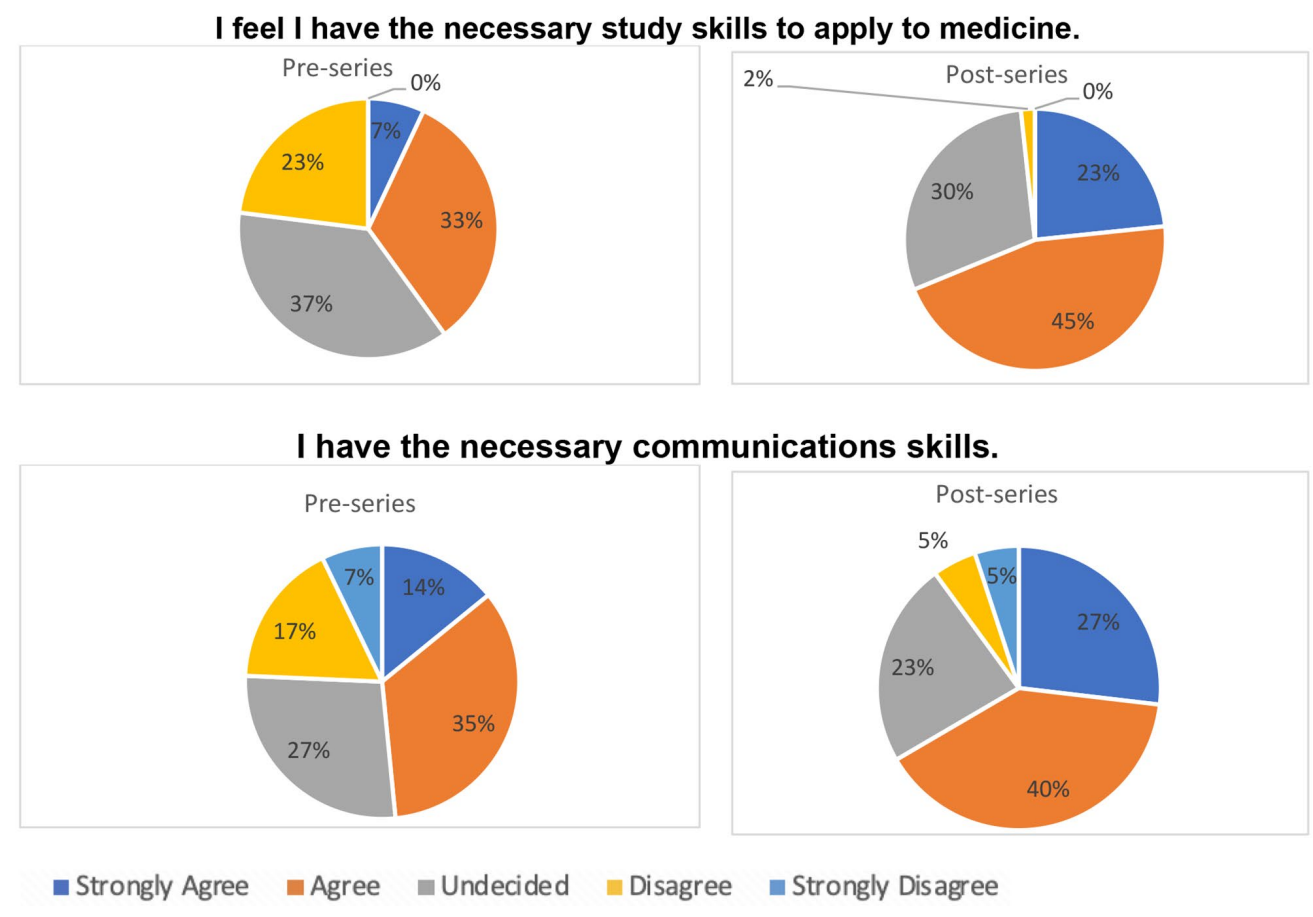
Pupil knowledge of the skills required in medicine pre- and post-series

The PAS series significantly increased pupils’ beliefs that they had the necessary skills to apply to medicine (*p* < 0.0001) and that they had the necessary communication skills to study medicine (*p* < 0.0001) (Fig. 2).

### Attitude

**Fig. 3:**
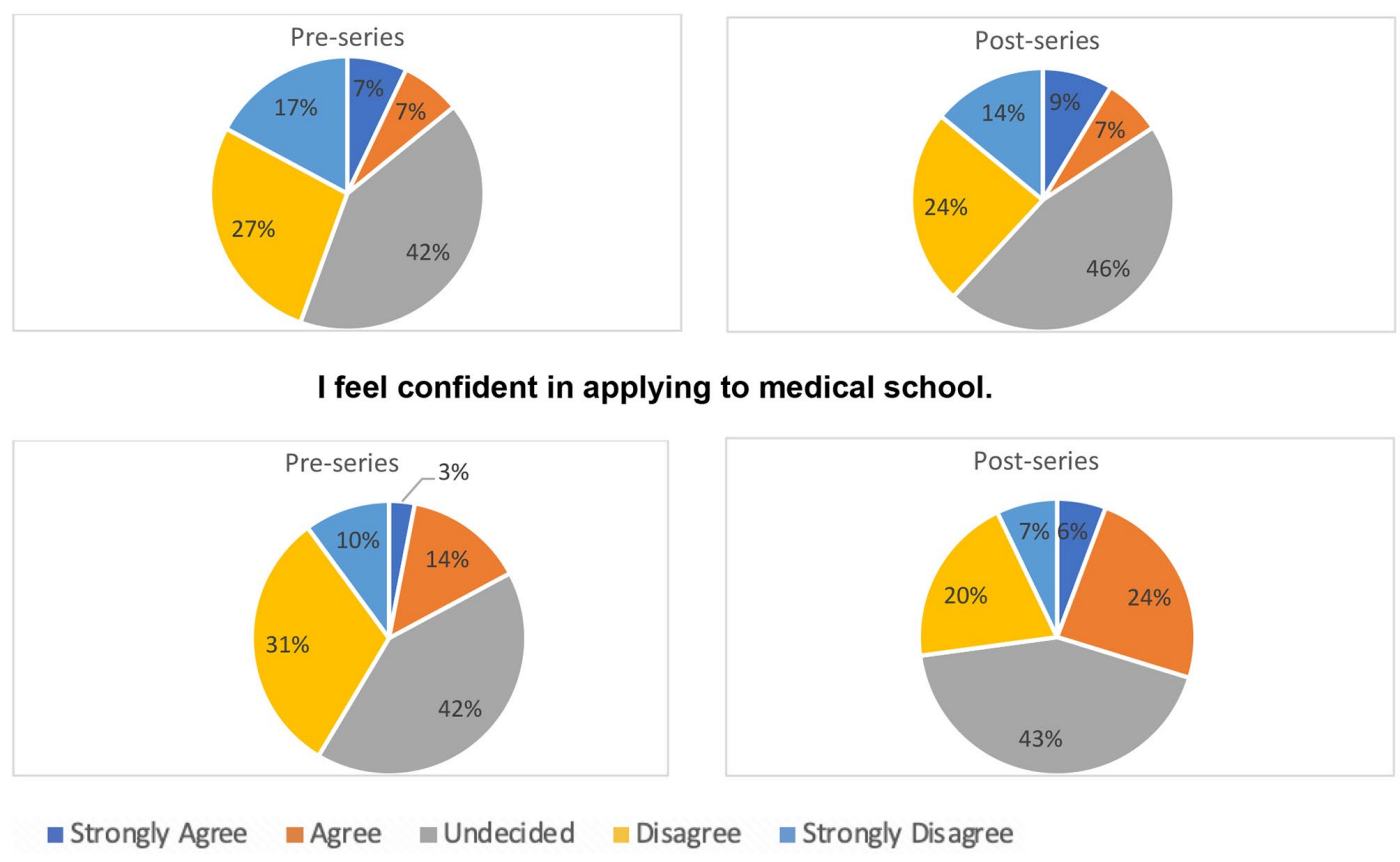
Pupils’ attitude towards medicine pre- and post-series

On analysis of pupils’ attitudes towards medicine, this study found a significant improvement in pupils’ confidence in applying to medical school after completing the PAS series (*p* < 0.0001). There was an improvement in pupils that wished to study medicine, but this was not statistically significant (*p* = 0.187). The proportion of pupils strongly agreeing to want to study medicine only rose from 7 to 9% upon completion of the PAS series; however, few pupils strongly disagreed or disagreed with wanting to study medicine after the series (Fig. 3).

### Pupils’ perceptions

To gain further insight in understanding factors that contributed to the changes in pupils’ perceptions in time regarding medicine as a degree and career, qualitative results were also collected. This provided opportunities to explore pivotal domains to consider and address when planning WP initiatives like PAS. A thematic analysis of open-ended comments provided by pupils on their perceptions of medicine as a degree and career on completing the PAS series is shown in Table 2. Many pupils mentioned they felt more informed after the series “I feel more informed about a career and how to study medicine because I have been told what I could take for GCSE and the steps and layout there is for applying to Medical School, which gives me a brief outline, therefore, I feel more confident”.

**Table 2:** Comments on changes to pupils’ perceptions of medicine as a degree and career

Theme*	Frequency
**Increasing knowledge** • “I have learnt that there is a lot more than just grades to studying medicine.” • “I have been provided resources that show me what the skills I need are and how I should approach my future studies. • “I think that this series has educated me much more on medicine, including the different options on studying medicine, what life would be like and how I could get to a medical career, however it has not convinced me to become a doctor.” • “I know now if I wanted to pursue an education or acquire an occupation as a doctor I would now the basics of how to do so.” • Informed me about a wider variety of medical careers and what the route into them may be, but not all people will have the same path.” • “I feel more informed about a career and how to study medicine because i have been told what I could take for GCSE and the steps and layout there is for applying to Medical School, which gives me a brief outline therefore I feel more confident.” • “I have been provided resources that show me what the skills I need are and how I should approach my future studies. • “We got to hear from professionals which widened my knowledge from people who have went through the process of medical school and becoming a doctor.”	32
**Reconsideration of Medicine** • “I used to want to cook now I want to study medicine to help people.” • “I’m now reconsidering about medicine if my other job doesn’t work out because I didn’t know that medicine was like a puzzle, and you have to try and figure out what is wrong with them and give them the right treatment.”	5
**Increased interest in Medicine** • “It sounds more interesting than I thought it would be.” • “I have seen that it isn’t just about working but also helping others, getting to make new relationships and it also seems quite fun.” • “Because you explained it in greater detail and in many ways to keep me engaged.”	3
**No changes** • “I don’t feel more informed, sorry.”	3
**Demotivation** • “The plant a seed series has informed me more about the subjects I would need to take to apply to medicine and about the life choices I would need to take and prepare myself for in order to become a doctor, however this series has demotivated me to become a doctor because I feel as though I lack the necessary skills to apply to medical school and study the required material.”	1
**Uncertainty** • “Not sure, don’t know if want to study medicine.” • “I have realised that the things that I am interested in could lead me to a career in medicine but I’m not sure if it’s for me.” • “The Plant a Seed series has definitely made me more aware of the options within the Medicine degree, but I couldn’t say 100% that I would go into medicine.”	1

*Only main comments are displayed to avoid repetition.

Other pupils felt they were now more aware of the different options within Medicine “The Plant a Seed series has definitely made me more aware of the options within the Medicine degree, but I couldn’t say one hundred per cent that I would go into medicine”. Some pupils expressed the PAS series did not change their perception of medicine.

The PAS series appeared to change some pupils’ perception of what a medical career entailed **“**I’m now reconsidering medicine if my other job doesn’t work out because I didn’t know that medicine was like a puzzle, and you have to try and figure out what is wrong with them and give them the right treatment”.

## Discussion

This study aimed to explore the impact of a low-cost online WP initiative on pupils’ knowledge, skills and attitudes toward a career in medicine. It found significant improvements in all three domains, demonstrating the effectiveness of the PAS series. Findings from this study emphasises the impact Universities can have and outlines the importance of prioritising widening participation at an early stage. PAS demonstrates the impact low-cost interventions can have and prioritisation of Government support through funding and measures such as annual social mobility league tables to encourage University involvement is key to bridge the gap and enable equal opportunities for all pupils [22].

However, the PAS study did not find that the intervention significantly increased pupils’ desire to study medicine. It is hard to gauge why this discrepancy took place. The written comments provided in the post-series survey suggested pupils’ knowledge of medicine as an undergraduate degree and career increased but they were still not sure if medicine was a career for them. This suggests that the PAS series may have allowed pupils to make informed decisions on their future. It is imperative to note that WP initiatives do not pressure pupils into applying to medicine but provide them with the level of knowledge and the necessary support to allow them to make an informed decision about their future degrees and careers. The pupils feedback suggested that this goal was achieved. On the other hand, other unknown factors may have contributed to the lack of significant change in pupils’ attitudes, which would need to be explored using qualitative methods. A potential factor that may have contributed to this could be the costs associated with an undergraduate medicine degree in the UK and the minimum five-year duration of the undergraduate degree before working and receiving a salary. Costs associated with undergraduate medicine for a UK student include tuition fee costs at £9,250 per year, living costs at an average £11,484 in year one and £12,350 for the following years, travel costs associated placements, equipment costs such as a stethoscope and costs for elective placements [23, 24]. This gives an estimated minimum required cost of £107,134 for tuition fees and living costs alone without the added interest and additional expenses mentioned. Thus, although pupils are now more aware of the programme through PAS, studying a medicine degree may not be a sustainable option for those in particular from lower socioeconomic backgrounds. Additionally, although PAS provides a deeper understanding of medicine as a degree and career at an early stage, support for more specific preparations for medicine applications such as the University Clinical Aptitude Test (UCAT) or BioMedical Admissions Test (BMAT) is still required depending on the universities applied for [4, 5]. Career barriers which may deter pupils from pursuing a career in medicine includes the debt associated with the medical degree, costs associated with training, long working hours and the salary in foundation training. A survey published by the British Medical Association in February 2023 found junior doctors financially struggling with over 50% of their utility bills and the current level of pay being among the top reasons for the consideration of leaving their role [25].

On the whole, the PAS series could adapt to having a focus on promoting medicine as a career. One suggestion would be to offer interactive Q&A sessions, as an additional component of the series, with the opportunity for pupils to ask a panel of doctors, academics and medical students any pressing questions they had about a medical career. A study found interaction during online sessions resulted in behavioural and emotional engagement with online learning and encouraged learning outside of the session [26]. However, the incorporation of live sessions would prevent the provision of a fully asynchronous programme and would need adequate resourcing.

### Strengths and limitations

A key strength of the study was the novelty of the intervention in its focus on Years 7–9 pupils from WP postcodes. As an online asynchronous intervention, PAS was delivered at a low cost to both schools and the university with no financial cost to pupils. This suggests a sustainable future for the intervention. We maintained an inclusive approach to recruitment, by ensuring the series was delivered during school hours. This meant that all pupils could access the videos. Pupils may not have access to the appropriate technology to run the series at home, given the known digital divide in the UK, with a recently estimated over 700,000 pupils having limited access to online learning outside school hours [27].

A key limitation was the small sample size given out of the 503 WP schools invited only 2 schools participated in the series. Contributing factors included the significant delay with ethics approval, which resulted in a short time to advertise and recruit schools. Another factor was that schools may have been put off by the need for pupils to view three 30–60-minute videos over the course of the academic year. This challenge was worsened by reduced teaching time, due to the national teachers’ strikes in 2023. Another barrier was the administrative burden of the study for each school, with paper consent forms and questionnaires adding to the high level of paperwork. Attempts to overcome this included the provision of a prepaid envelope and an offer to provide printed forms to avoid the need to scan in the signed forms. However, we did not have a solution for reducing the time to collect all consent/assent forms from pupils.

### Future work

Future analysis at scale and over a longer period of follow-up will be undertaken to fully capture the impact of this series on pupil’s knowledge, skills and attitudes to medicine as a degree and career and quantify the number of pupils who applied to and were successful on the medicine programme. We propose to use HEAT tracking to establish the long-term impact of the PAS series. HEAT tracking would enable them to see how many of the pupils who completed PAS entered medical school [21]. However, the team accept that PAS would only be one contributing factor to a pupil deciding on a career in medicine. Analysis at scale would also enable a sufficient sample size for sub-group analysis of the impact of gender, age, ethnicity, parental occupation and socioeconomic status on the overall questionnaire score. We suggest a future study should incorporate rigorous qualitative analysis with qualitative comments [28]. This data could be obtained through ethics-approved focus groups of pupils that participated in the study.

We also plan to discuss PAS with schools to explore how they can make the process easier for the teachers and administrative staff. This would increase uptake and help increase the generalisability of study findings to areas across the UK. Although a strength of the study included enrolment through schools and not pupils, a limitation was that it meant that pupils potentially interested in PAS could not enrol without interest from their school. Future work could include recruiting pupils directly. However, pupils from WP backgrounds may not have access to facilities at home to view the videos, and it may be cumbersome obtaining consent for them.

## Conclusion

Findings from this study underscore the impact low-cost and effective WP interventions like PAS can have on significantly improving pupils’ knowledge, skills and confidence in considering a degree and career in medicine and evidences the need for government and academic institutions to invest in and award such interventions. PAS will be delivered on a national scale to schools in deprived areas of the UK to allow pupils to make more informed decisions about their future and will improve inclusivity and diversity in the healthcare workforce.

## Data Availability

The datasets used and/or analysed during the current study available from the corresponding author on reasonable request.

## Abbreviations

HEAT: Higher Education Access Tracker
PAS: Plant a Seed
WP: Widening participation
